# Hidden diversity in Antarctica: Molecular and morphological evidence of two different species within one of the most conspicuous ascidian species

**DOI:** 10.1002/ece3.6504

**Published:** 2020-07-15

**Authors:** Micaela B. Ruiz, Anabela Taverna, Natalia Servetto, Ricardo Sahade, Christoph Held

**Affiliations:** ^1^ Instituto de Diversidad y Ecología Animal (IDEA) Consejo Nacional de Investigaciones Científicas y Técnicas (CONICET) Córdoba Argentina; ^2^ Facultad de Ciencias Exactas Físicas y Naturales Departamento de Diversidad Biológica y Ecología, Ecología Marina Universidad Nacional de Córdoba Córdoba Argentina; ^3^ Section Functional Ecology, Evolutionary Macroecology Alfred Wegener Institute Helmholtz‐Zentrum für Polar‐ und Meeresforschung Bremerhaven Germany

**Keywords:** Antarctica, Burdwood Bank/MPA Namuncurá, *Cnemidocarpa verrucosa*, genotypes, morphotypes, species delimitation

## Abstract

The Southern Ocean is one of the most isolated marine ecosystems, characterized by high levels of endemism, diversity, and biomass. Ascidians are among the dominant groups in Antarctic benthic assemblages; thus, recording the evolutionary patterns of this group is crucial to improve our current understanding of the assembly of this polar ocean. We studied the genetic variation within *Cnemidocarpa verrucosa* sensu lato, one of the most widely distributed abundant and studied ascidian species in Antarctica. Using a mitochondrial and a nuclear gene (COI and 18S), the phylogeography of fifteen populations distributed along the West Antarctic Peninsula and Burdwood Bank/MPA Namuncurá (South American shelf) was characterized, where the distribution of the genetic distance suggested the existence of, at least, two species within nominal *C. verrucosa*. When reevaluating morphological traits to distinguish between genetically defined species, the presence of a basal disk in one of the genotypes could be a diagnostic morphological trait to differentiate the species. These results are surprising due to the large research that has been carried out with the conspicuous *C. verrucosa* with no differentiation between species. Furthermore, it provides important tools to distinguish species in the field and laboratory. But also, these results give new insights into patterns of differentiation between closely related species that are distributed in sympatry, where the permeability of species boundaries still needs to be well understood.

## Graphical abstract

The evidence presented here allowed to delimitate two species within the conspicuous Antarctic ascidian *C. verrucosa*: two monophyletic groups were obtained, and congruent mitochondrial/nuclear species delimitation was demonstrated. Our results once again emphasize that species identities, even for highly abundant and well‐studied species on small local scales, must be assessed rather than assumed.

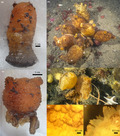

## 1 INTRODUCTION

Triggered by a steep decline in atmospheric CO_2_, the Antarctic polar front (APF) has been functioning as a geographic, climatic, thermal, and oceanographic barrier that isolated the Southern Ocean from lower latitude surface waters since the Eocene/Oligocene boundary (DeConto & Pollard, 2003). The Antarctic circumpolar current (ACC) has also played a role as an isolating force around Antarctica reducing the southward oceanic heat transport since Drake Passage opening (Martinson, 2012; Rintoul, Hughes, & Olbers, 2001). As a consequence, the continent of Antarctica suffered a series of glaciation cycles that fragmented its marine biota on the Antarctic shelf (Cristini, Grosfeld, Butzin, & Lohmann, 2012; Hewitt, 2004; Petit et al., 1999; Soler‐Membrives, Linse, Miller, & Arango, 2017). The Southern Ocean is one of the most diverse and rich marine ecosystems with a high level of endemism, even in comparison with temperate and tropical environments (Allcock & Strugnell, 2012; Halanych & Mahon, 2018; Rogers, 2007). Numerous cryptic species were discovered in this region, that is, genetically distinct species that have been previously classified as a single species due to their similar phenotypes (Bickford et al., 2006; Held, 2003; Held & Wägele, 2005). Therefore, the real species number in Antarctica may be significantly higher than the numbers of formally described species currently suggest; thus, species yet undescribed represent an important portion of the true biodiversity (Dömel et al., 2015; Galaska, Sands, Santos, Mahon, & Halanych, 2017; Havermans, Nagy, Sonet, De Broyer, & Martin, 2003, 2011; Riesgo, Taboada, & Avila, 2015; Wilson, Hunter, Lockhart, & Halanych, 2007).

In order to understand the evolution of biodiversity, it is necessary to recognize species. A unified species concept has to deal with the problem of inferring the boundaries of species, and concepts such as cryptic and pseudocryptic species that still need to be well attended. An interdisciplinary approach that involves combining data on genomic and phenotypic traits is necessary to start solving this problem (Heethoff, 2018; Struck et al., 2018). Molecular studies have revealed complexes of cryptic species in ascidians, such as *Ciona intestinalis* (Caputi et al., 2007), *Botryllus schlosseri* (Bock, Macisaac, & Cristescu, 2012; Nydam, Giesbrecht, & Stephenson, 2017; Yund, Collins, & Johnson, 2015), and *Diplosoma listerianum* (Pérez‐Portela, Arranz, Rius, & Turon, 2013). However, only a small subset of these MOTUs (molecular operational taxonomic units) have been verified using morphological characters, for example, in the cryptic species complex *Ciona intestinalis* (Brunetti et al., 2015).

Ascidians are an important group in the Antarctic benthic communities, being even dominant in some assemblages (Gili et al., 2006; Sahade et al., 2015; Tatian, Sahade, Doucet, & Esnal, 1998). The genus *Cnemidocarpa* (Huntsman, 1913) is one of the most rich in species in the Antarctic and Sub‐Antarctic areas, comprising more than 10 described species (Monniot & Monniot, 1983). This genus is characterized by the shape of the gonads: They are more or less elongate, often tubular, occasionally branched, and few in number (one or two on each side of the body), and the ovary and testes are in close contact with each other and enclosed in a sheathing membrane. *Cnemidocarpa verrucosa* (Lesson, 1830) (Chordata, Tunicata) is the largest and most abundant styelid in the Antarctic Ocean. It can inhabit muddy to hard bottoms and waters between five and more than 770 m deep (Monniot, Dettai, Eleaume, Cruaud, & Ameziane, 2011; Ramos‐Esplá, Cárcel, & Varela, 2005; Tatian et al., 1998). *Cnemidocarpa verrucosa* is a solitary broadcasting ascidian, possessing lecithotrophic larvae and strong seasonality in reproduction (Bowden, Clarke, & Peck, 2009; Sahade, Tatián, & Esnal, 2004; Strathmann, Kendall, & Marsh, 2006). This species was described in Malvinas/Falkland Islands by Lesson (1830), but was later also widely reported from the Antarctic continental shelf and is considered to have a circumpolar distribution in the high Antarctic as well as the sub‐Antarctic (Herdman, 1881; Kott, 1969; Michaelsen, 1898; Monniot & Monniot, 1983; Monniot et al., 2011; Sluiter, 1905; Turon, Cañete, Sellanes, Rocha, & López‐Legentil, 2016, 2016).

Considering the pervasive discovery of cryptic species in the Southern Ocean and elsewhere, the goals of this work were (a) to determine whether there are more than one genetically divergent species within the nominal *C. verrucosa*; (b) to resolve whether the presumable species are also morphologically distinguishable; (c) to test whether species within *C. verrucosa* co‐occur; and if so, to test whether their co‐occurrence can be explained by secondary contact. Furthermore, being able to discriminate species without having to rely on molecular results in the laboratory and also in the field may have implications in many research fields, especially in biodiversity and experimental studies.

## 2 MATERIALS AND METHODS

### 2.1 Sampling

**Figure 1:**
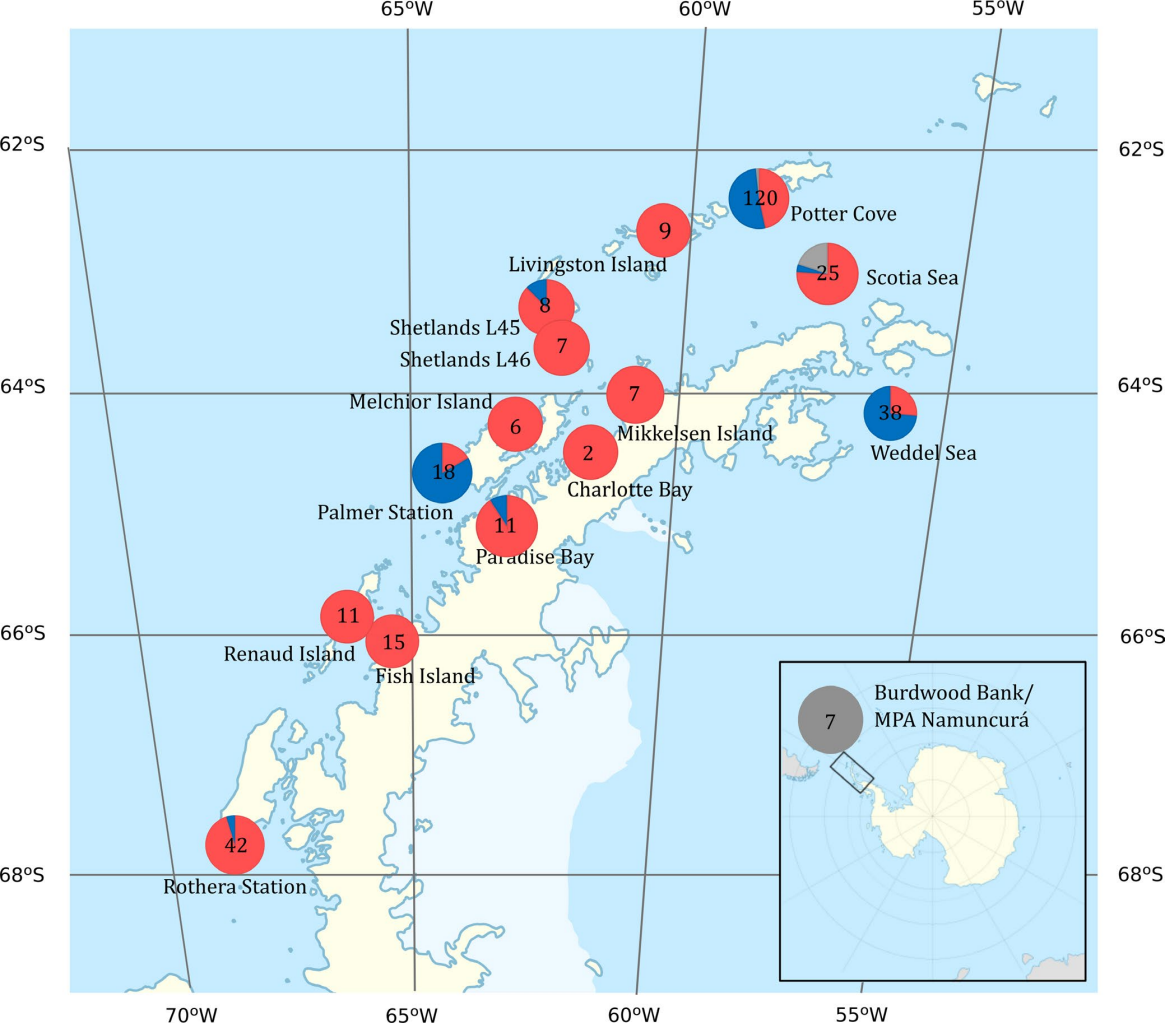
Species distribution along the WAP. Each circle represents a sampling station, the proportion of *Cnemidocarpa verrucosa* sp. A in red, the proportion of *C. verrucosa* sp. B in blue, and no A/no B samples sequences only with 18S in gray (basal branch, groups C‐F in ABGD analysis). Numbers represent sample size

Samples for genetic analysis were collected during different campaigns: (a) *Mission Antarctique* campaign, on board the “R/V Sedna IV” in 2006; (b) Summer Antarctic Campaign at Potter Cove (Carlini Station, King George Island, Antarctica) in 2007/2008; (c) BENTART‐06, on board the “B.I.O. Hespérides” in 2006; (d) ANT XXIX/3 in 2013 on board the “R/V Polarstern”; and (e) PD BB April 17, on board the “R/V A.R.A. Puerto Deseado” to Burdwood Bank/MPA Namuncurá 2017 (MPAN‐BB). During campaigns, a and b samples were obtained by SCUBA diving, while in campaigns c, d, and e samples were obtained by bottom trawls (see depth of sampling in Table S1). Fourteen stations were sampled along the West Antarctic Peninsula (WAP) and one in South America in MPAN‐BB (Figure 1), the naming of sampling stations follows the SCAR Composite Gazetteer of Antarctica (1992, updated 2020). Mantle tissue (of approximately 1 cm^3^ size) dissected from specimens for genetic analysis was conserved in denatured ethanol 96% (Sigma‐Aldrich) until DNA extraction. To obtain entire animals was not possible in the framework of campaigns a, b, c, d, and e.

Samples for morphological analyses (i.e., entire animals) were collected in January 2018 by SCUBA diving between 20 and 30 m depth at Potter Cove (Carlini Station, King George Island, Antarctica). Specimens were relaxed using menthol crystals (TodoDroga), animals were placed in big trays and submerged in seawater, menthol crystals were placed inside the trays, and after two hours, a probe was inserted into an open siphon to check whether there was absolutely no response. If there was still a response, the animals were kept there for another hour. Once complete relaxation was achieved, the animals were fixed in denatured ethanol 96%. The examined material for morphological analyses is stored in the collection of the Instituto de Diversidad y Ecología Animal, IDEA, CONICET y UNC.

### 2.2 DNA extraction, PCR and sequencing

Total DNA was extracted from up to 25 mg mantle muscle tissue using the DNeasy Mini Kit (Qiagen) according to the standard tissue protocol but reducing the final elution volume to 100 μl. In order to exclude contamination with DNA from other organisms or food, dissection was carried out using sterilized forceps and scalpels, isolating the mantle from the rest of the tissues such as intestine and tunic.

#### 2.2.1 Cytochrome c oxidase subunit I (COI) PCR

The tunicate primers pair Tun_reverse2 (Rev) (Stefaniak et al., 2009) and Cve‐CO1‐F54 (Fwd) 5′ AGTGTTTTAATTCGAACAGA 3′, and the primers pair Deg COI F2 (Fwd) and Deg COI R2 (Rev) (Reem, Douek, Paz, Katzir, & Rinkevich, 2017) were used for amplification. The primer Cve‐CO1‐F54 (Fwd) was designed within this study due to the bad quality (double peaks, ill‐defined or garbled peaks in the chromatograms) of forward sequences obtained with the Stefaniak‐primer Tun_forward. The primer Cve‐CO1‐F54 (Fwd) was designed using the software Geneious version R8 (Kearse et al., 2012) and based on good quality forward sequences from this work. Reactions were carried out in 25 μl volumes, using 0.025 U/µl of Promega GoTaq G2 Flexi DNA Polymerase, 30 ng of DNA, 0.5 µM of each primer, and 2 mM of MgCl_2_. The amplification protocol was 2 min at 94°C for initial denaturation followed by 36 cycles of 60 s at 94°C, 50 s at 46°C, 50 s at 72°C, and a final elongation step of 8 min at 72°C.

#### 2.2.2 Nuclear Ribosomal RNA Gene (18S rDNA) PCR

Primers 18S1 (Fwd) and 18S4 (Rev) (Tsagkogeorga et al., 2009) were used for amplification. Amplifications were carried out in 25 μl volumes, using 0.03 U/µl of TaKaRa LA Taq HS, 30 ng of DNA, 0.5 µM of each primer, and 0.05 mM of Betaine. The amplification protocol was 1 min at 94°C for initial denaturation followed by 30 cycles of 10 s at 98°C, 50 s at 50°C, 2 min at 72°C, and a final elongation step of 10 min at 72°C.

PCR products were visualized on a 1% TAE agarose gel stained with GelRed (Nucleic Acid Gel Stain) under UV illumination. PCR products were outsourced for sequencing to Eurofins MWG Operon (Germany) on an ABI3730XL automatic DNA sequencer, using either of the two terminal primers used for amplification.

### 2.3 Sequence alignment and phylogenetic analysis

Nucleotide sequences were edited, assembled, and aligned using the program Codon Code Aligner (v. 5.1.5, Codon Code Corporation) with the Muscle plugin (Edgar, 2004). Primer sequences used for amplification were excluded from the analysis, and COI sequences were translated into amino acid sequences based on the Ascidian mitochondrial code (translation table 13) to further improve sequencing quality and screen for frameshift mutations and stop codons.

Genetic polymorphism analysis was run for each population calculating the number of haplotypes (Nh), haplotype diversity (h), and nucleotide diversity (π) using DnaSP version 5.10.01 (Librado & Rozas, 2009). Sequences of 18S rDNA were phased with the PHASE v2.1.1 algorithm (Stephens & Donnelly, 2003; Stephens, Smith, & Donnelly, 2001) in DnaSP using default parameters. Pairwise *F*_ST_ among all populations and AMOVA were calculated using ARLEQUIN v. 3.5.2.2 (Excoffier, Laval, & Schneider, 2005). The significance of the variance components and pairwise *F*_ST_ values were assessed by a permutation test with 10,000 replicates. To test isolation by distance in *C. verrucosa* populations, a Mantel test with 1,000 permutations was performed using the IBD Macintosh application v. 1.52 (Bohonak, 2002). Scatter plot of geographic distance and genetic distance was performed in R v3.6 for Microsoft Windows (R Core Team, 2020). The genetic distances among populations were expressed as *F*_ST_ pairwise differences. The geographic distances between populations were represented by the shortest coastline distance.

Species delimitation was carried out using the online version of Automatic Barcode Gap Discovery, ABGD (http://wwwabi.snv.jussieu.fr/public/abgd/) using Kimura p‐distance. ABGD delimits a “barcode gap” in the distribution of pairwise differences (Puillandre, Lambert, Brouillet, & Achaz, 2012). The haplotype network was created with Haploviewer (available at www.cibiv.at/~greg/haploviewer), based on multiple alignments of the sequences and on a neighbor‐joining tree that was constructed using the software MEGA 7.0.21 (Kumar, Stecher, Li, Knyaz, & Tamura, 2018).

For phylogenetic reconstruction, the most suitable model of molecular evolution was determined from the data with the software jModeltest 2.1.9 v20160115, with 88 candidate models, using Bayesian information criterion. The best‐fit model for COI was HKY85+G+ I, and for 18S, the best‐fit model was HKY85+G+I; these models were applied in maximum likelihood (ML) and Bayesian inference (BI) analyses. ML analysis was run using PhyML v.3.0 (Guindon et al., 2010) using 1,000 bootstrap replicates for both markers independently. BI analysis was run using Markov Chain Monte Carlo (MCMC) simulations in MrBayes v3.2 (Ronquist et al., 2012); sampling every 100 generations, samples of the substitution model parameters were checked whether the likelihoods reached stationarity, and whether the standard deviation of split frequencies was below 0.05. Mitochondrial COI reached stationarity after a total of 500,000 MCMC generations (split = 0.04), while 18S with a total of 200,000 MCMC generations (split = 0.02). The sampled trees were used to infer Bayesian posterior probabilities (BPP) for the nodes and produce the consensus tree.

In order to estimate divergence time since speciation, the BEAST 1.8.0 software package was used to analyze COI sequences (Drummond, Suchard, Xie, & Rambaut, 2012). First, xml files were generated using BEAUti to execute them in BEAST. Data from other marine invertebrates were used as a proxy since due to lack of adequate fossil records, and no calibrated mutation rates for ascidians for COI exist in the bibliography. Nydam and Harrison (2011) estimated from data based on other marine invertebrate taxa (crabs, shrimp, urchins), a mutation rate range of 0.016–0.026 substitutions per site per million years. Two independent analyses were run: a first one using strict clock model with a substitution rate of 0.016 substitutions per site/ million years (10^7^ generations), and a second one at 0.026 substitutions per site/ million years (10^7^ generations), and for both, a burn‐in of 20% was applied and discarded. The tree prior was set to Yule speciation. The GTR + G substitution model was used. The xml files were then executed in BEAST. Results were analyzed using Tracer v1.6.0 to check the convergence to a stationary distribution of parameters.

### 2.4 Morphological analysis

A total of 23 specimens assumed to be the putative *C. verrucosa* were examined for morphological analyses (see section 2.1). The samples were dissected, analyzed, and photographed using a stereoscopic microscope (Labomed CZM4 and CZM6) equipped with a digital camera for identification and documentation of internal characters. We analyzed the principal external and internal morphological characters for *Cnemidocarpa verrucosa* (Lesson, 1,830) following established procedures (Millar, 1960; Monniot & Monniot, 1983; Turon, Cañete, Sellanes, Rocha, & López‐legentil, 2016). The external characteristics measured were the following: (a) position of the siphons (both terminal siphons on the same line on the distal part, or one of them oriented toward one side), (b) presence of basal disk, (c) shape of warts (rounded and smooth, or conical with multiple spine‐like endings), (d) height, and (e) width. After dissection, following internal structures were noted: (a) number of oral tentacles; (b) total number of longitudinal vessels in folds of the branchial sac; (c) total number of longitudinal vessels between the folds of the branchial sac; (d) total number of gonads (left and right); and (e) number of stomach folds. Sequencing of COI and 18S markers was carried out for these individuals in order to test the relationship between genetic and morphological grouping.

### 2.5 Morphological data analysis

A mixed data matrix was created with all morphological characters analyzed. For multivariate analysis, a logarithmic transformation was made for quantitative variables (internal structures 1, 2, 3, 4, and 5). The height and width were not used for the analysis because these two characters are highly variable due to the elastic nature of the animal and the amount of water it contains. Nevertheless, correlation was tested among these two characters and the rest of the studied ones, and no significant relation was found (data not shown). Multivariate analyses were used to determine affinities between specimens of *Cnemidocarpa verrucosa* based on a morphological character matrix. Nonmetric multidimensional scaling (NMDS) was performed using three dimensions and Gower distance. Nonparametric multivariate analysis of variance (PERMANOVA) was used to assess differences between the groupings obtained in NMDS, and each term of the analysis was tested using 9,999 permutations. The software package PAST 3.16 was used for all the morphological data statistics (Hammer, Harper, & Ryan, 2001).

## 3 RESULTS

### 3.1 Mitochondrial Cytochrome c oxidase subunit I (COI) and Nuclear Small Ribosomal Subunit RNA Gene (18S rDNA)

**Figure 2:**
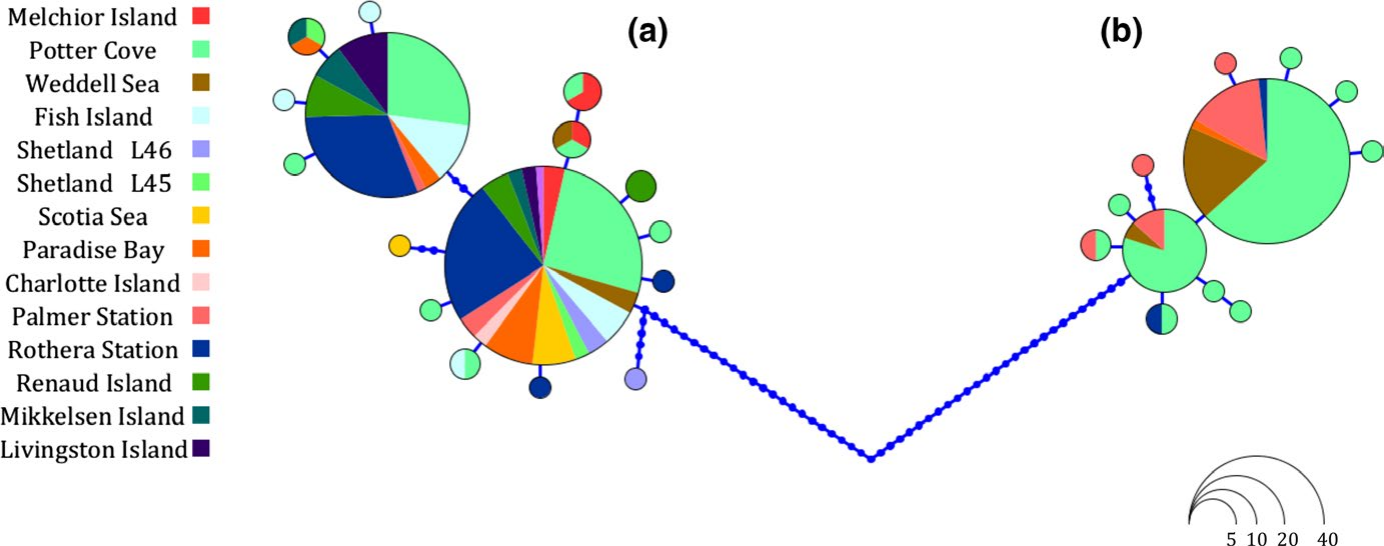
Haplotype network of COI mitochondrial gene. Areas of the circles are proportional to the number of individuals. Each circle represents a haplotype, and dots between haplotypes symbolize mutational steps

The aligned fragment of the COI gene was 503 bp long excluding the amplification primers, and in total, 253 individuals from 14 stations were sequenced successfully (https://doi.org/10.1594/PANGAEA.909707). The alignment does not contain gaps, and translation of sequences to amino acid sequences revealed no frameshift mutations or stop codons. The analysis of the sequences identified 28 haplotypes, 70 sites were polymorphic and 56 parsimony informative. The haplotype network (Figure 2) showed two sharply distinct groups separated by 50 mutational steps. Group A is distributed all along the WAP and shows two dominant haplotypes, Group B is distributed in Weddell Sea, Potter Cove, Palmer Station, Paradise Bay, and Rothera Station, again with two common haplotypes that are mainly present in Palmer Station. In addition, there are 18 rare haplotypes, represented by one or two individuals from a single location.

The aligned sequences from the 18S fragment, containing the V4 ribosomal expansion segment, were 877 bp long. In total, 312 individuals were sequenced, and the alignment contained no gaps (https://doi.org/10.1594/PANGAEA.909707). We found 70 polymorphic sites, all parsimony informative, and after phase haplotype reconstruction, 10 haplotypes were recognized. From the 70 polymorphic informative sites, a single site at position 444 contained two variants that were congruent with the division among the mitochondrial groups A and B (Figure 2). A single individual (collection code 291) showed both nucleotides (thymine and cytosine, respectively), and this can be interpreted as individual 291 being heterozygous or hybrid (see discussion section 4.4).

The highest haplotype diversity for COI was found in Potter Cove, but almost all the populations presented high values of diversity except for Livingston Island and the Scotia Sea. On the other hand, the highest haplotype diversity for 18S was observed in MPAN‐BB, but also Potter Cove, the Scotia Sea, and Shetland L45 and L46 presented high diversity values (Table S1). Sequencing of COI was not possible for some individuals (all individuals from MPAN‐BB, five individuals from Scotia Sea, and two from Potter Cove), several pairs of primers were tested (Bishop et al., 2013; Folmer, Hoeh, Black, & Vrijenhoek, 1994; Monniot et al., 2011), and with no successful amplification, possible reasons for this are discussed later (section 4.1).

### 3.2 Species delimitation

Automatic barcode gap analysis showed a bimodal distribution in COI sequences pairwise differences. The genetic distance within each group for COI was <1.41%, whereas the genetic distance among groups was >10.20% with no intermediate pairwise distances observed. ABGD analysis for 18S sequences distinguished six groups. Genetic distance between within groups was 0.00%. Two groups (named here A and B) are congruent with the groups already identified by the ABGD analysis of COI sequence data. The genetic distance between group A and B exceeded 0.11%, with no intermediate distances observed. Sequencing of COI for individuals assigned to groups C, D, E, and F was not possible (see section 4.1). The distance between group C and groups A‐B was > 2.79%; distance between group D and groups A‐C was > 4.85%; distance between group E and groups A‐D was > 4.11%; and distance between group F and groups A‐E was > 4.49%. This indicates that groups C, D, E, and F may correspond to other not sp. A or B, more distant, cryptic species.

### 3.3 Phylogenetic analysis and divergence time

**Figure 3:**
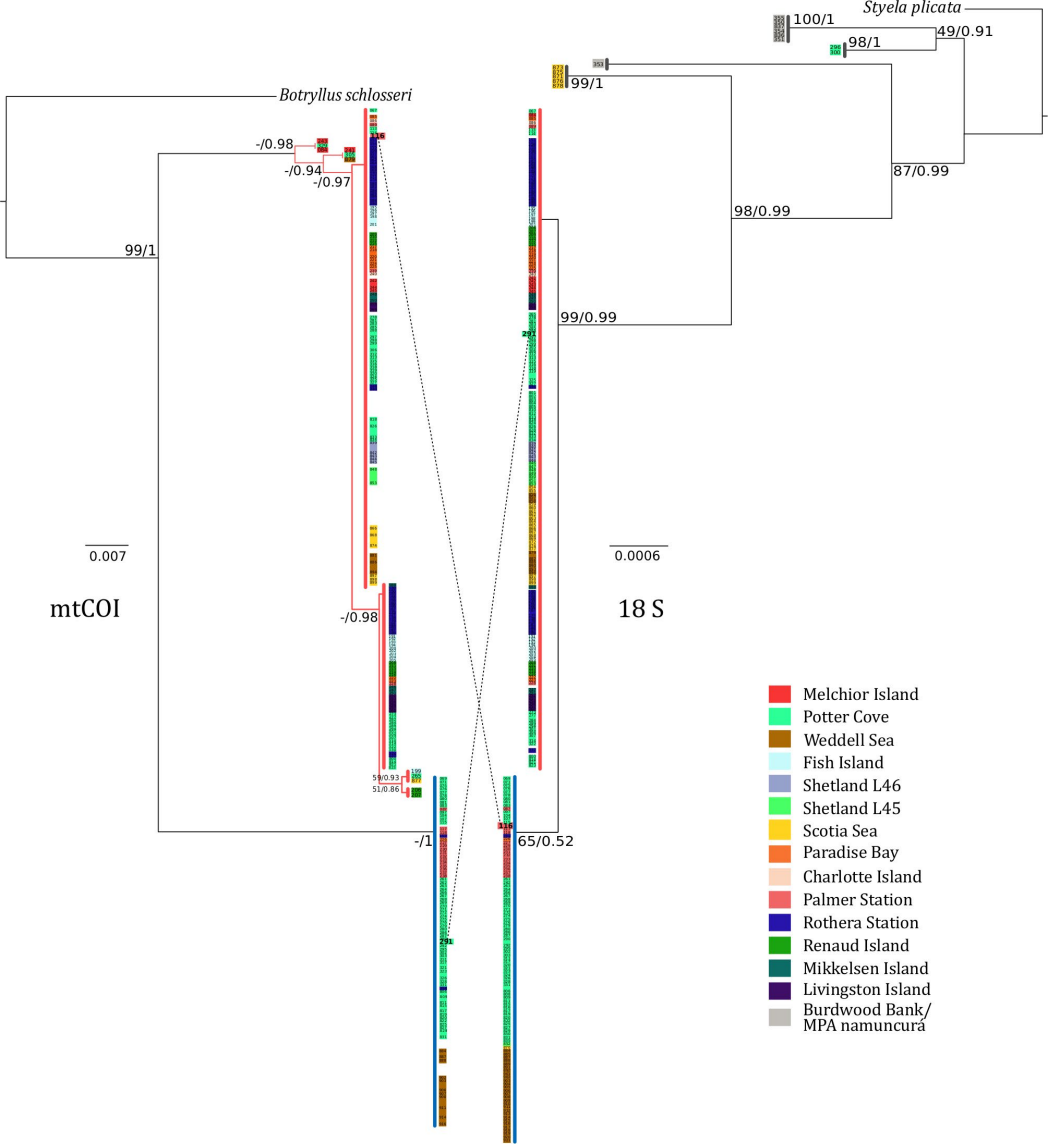
Phylogenetic trees obtained with maximum likelihood and Bayesian inference approaches. Left, phylogeny constructed with the mitochondrial marker COI. Right, phylogeny constructed with the nuclear marker 18S. Only nodes supported by bootstrap value ≥51 and posterior probability ≥0.86 are reported. Mirrored pattern of branching clades is observed, and the dotted lines indicate the only two samples that do not show this congruent pattern. Branches in red correspond to group A, and group B in blue defined by ABGD species delimitation analysis and the haplotype network of COI. Branches in gray correspond to groups C, D, E, and F of ABGD analysis. Each number represents one individual; background colors of numbers represent sampling stations. Note that not all the individuals were sequenced by both markers

The phylogenetic trees based on both molecular markers (nuclear and mitochondrial), and using a maximum likelihood and Bayesian inference approach shows two well‐supported, reciprocally monophyletic, and congruent groups of samples (posterior probability, PP ≥ 0.99; bootstrap probability, BP = 99) (Figure 3). Group “A” occurred on all stations along the WAP, whereas group “B” was missing in many sampled stations (Figure 3). Moreover, the phylogeny built with the nuclear gene separated four extra groups congruent with groups C–F delimited in ABGD analysis (in gray in Figure 3). One cluster comprises exclusively individuals from MPAN‐BB (PP 1, BP 100; group D in ABGD analysis), a second group with two samples from Potter Cove (PP 1, BP 98; group E in ABGD analysis), a third branch with only one sample from MPAN‐BB; group F in ABGD analysis, and a fourth group constituted by samples from Scotia Sea (PP 1, BP 99; group C in ABGD analysis).

The two congruent clusters defined by the nuclear and mitochondrial phylogenetic trees were grouping the sequences in the same way as ABGD analysis (groups A and B) and the haplotype network. However, two individuals were assigned to different groups depending on which marker was used, mitochondrial or nuclear. On the one hand, individual 291 from Potter Cove was assigned to group A according to 18S species delimitation, but to group B in COI. On the other hand, the individual 116 from Palmer Station was assigned to group B in 18S species delimitation, but to group A for COI. This crossed pattern is incompatible with congruent diversification of mitochondrial and nuclear genes, and possible explanations are discussed below (see discussion section 4.4).

The estimation of divergence time among groups A and B, using COI sequences, was calculated between 3.58 Ma (95% high posterior density [HPD]: 2.331–4.935 Ma) and 2.20 Ma (95% high posterior density [HPD]: 1.423–3.028 Ma).

### 3.4 Morphological analysis

**Figure 4:**
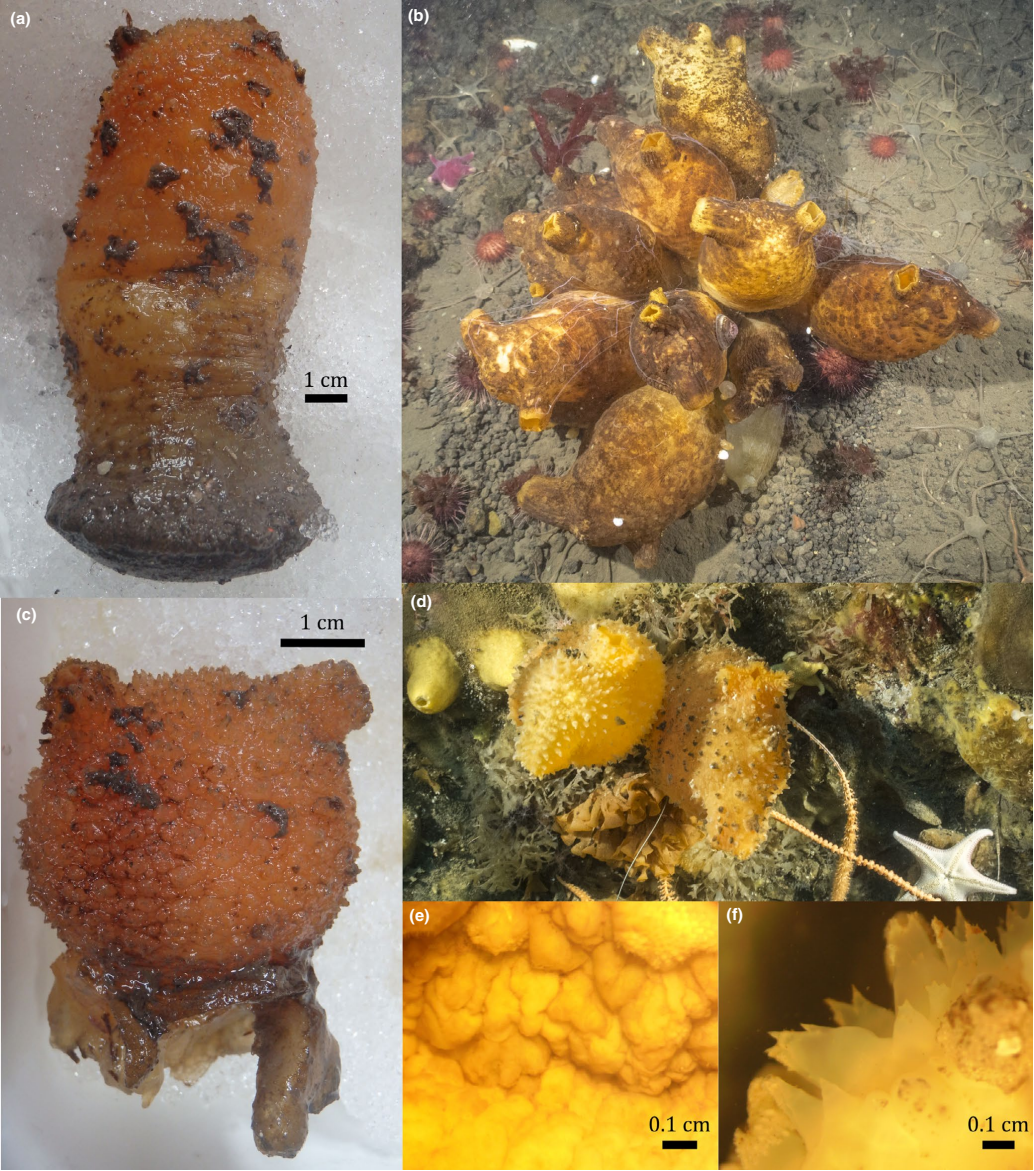
Photographs. (a) specimen with basal disk, and (b) specimen without basal disk. To the right (c and d) underwater photographs taken by Cristian Lagger, in the benthos assemblage of Potter Cove where high diversity of morphological features is observed *in Cnemidocarpa verrucosa* sensu lato

The appearance of this species is characteristic: large, robust body, ovate, or ellipsoidal. Usually, it is not compressed laterally. Specimens analyzed were all sexually mature and varied between 5.8 and 17.3 cm in length, and 4.1 and 9.6 cm in width. Siphons were located in the anterior part of the body; from 25 specimens, only eight had siphons with different height. Ten specimens had a basal disk, and to define “basal disk,” we followed Kott (1971) descriptions of *C. verrucosa*. According to her work, the animal is attached to the substrate by way of a “stalk” that is expanded toward its base, the body wall prolongs into a muscle‐free jelly‐like extension that expands into a basal plate in the base of the stalk, and this structure increase in thickness to form a sort of spherical rhizome constricted off from the rest of the body. According to the description the tunic, although hard, is usually quite thin and somewhat soft and flexible, orange, brown or yellow in live specimens. In fixed specimens, the tunic was yellowish, brown, or gray. *Cnemidocarpa verrucosa* is characterized by the presence of warts in the tunic. In the present study, some specimens showed rounded and smooth warts, others presented conic warts with multiple spine‐like ends, and some showed both types distributed in diverse ways on the tunic with no clear pattern (Figure 4). Internal characteristics represented the intraspecific variation previously described for this species: the branchial sac had fourfolds in each side of the body, the number of longitudinal vessels in folds of the branchial sac ranged from 7 to 21, and the number of longitudinal vessels between the folds of the branchial sac ranged from 1 to 5. The oral tentacles are filiform, alternating in size (short and long), and the number ranged from 22 to 38. No siphonal spinules were found. The intestine was located on the middle ventral left side of the body, and there are 19–30 stomach folds. The gonads were tubular, testes in the core and ovaries enclosing it. Most specimens had two gonads at each side of the body; nevertheless, specimens cv12 and cv16 had two on the right side and one on the left side, and cv23 showed one gonad on each side and cv25 two gonads in the right side. The distal end with gonoducts was directed toward the atrial siphon (raw data in https://doi.org/10.1594/PANGAEA.909707).

**Figure 5:**
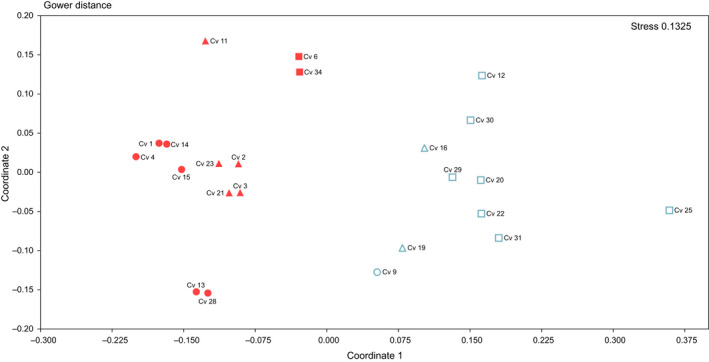
Nonmetric multidimensional scaling of the morphological characteristics of *Cnemidocarpa verrucosa* specimens from Potter Cove. Numbers refer to different individuals analyzed. Color red indicates the genetic species *C. verrucosa* sp. A, and blue indicates *C. verrucosa* sp. B. Shape of symbols indicates of type of warts: circle: rounded and smooth, triangle: conic with multiple spine‐like ends, and square: conic and smooth to ventral, smooth toward the siphons. The filled symbols indicate the absence of basal disk, and the empty symbols indicate the presence of basal disk

The NMDS showed two groups among samples (Figure 5). The two groups identified in the NMDS coincided with the specimens genetically identified as groups A and B in the genetic analyses. PERMANOVA revealed significant differences between the groups conformed in NMDS analysis (*F* = 17.17; *p* = .0001). All specimens from group B had a basal disk, while none of the specimens from group A did. None of the other morphological characters analyzed in this study appeared to be phylogenetically informative.

### 3.5 Population structure

Given the results from genetic and morphological species delimitations (see above), it is highly likely that the two groups that were congruently identified in nuclear, mitochondrial, and morphological characters correspond to two reproductively isolated species inside nominal *Cnemidocarpa verrucosa* sensu lato, which in the following will be called *C. verrucosa* sp. A and *C. verrucosa* sp. B. Hence, the following analyses were carried out separately for each delimited species of *C. verrucosa* sensu stricto.

**Figure 6:**
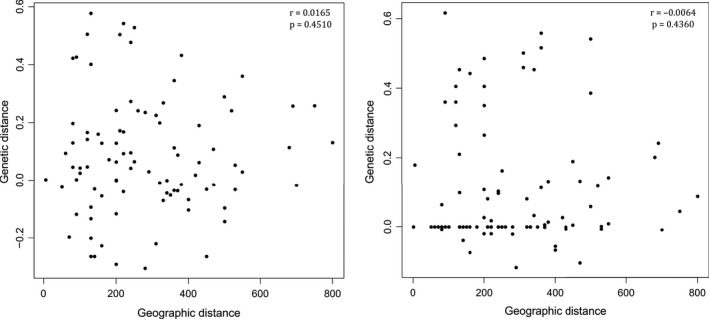
Isolation by distance analysis. *Cnemidocarpa verrucosa* A pairwise genetic distances (*F*_ST_) and geographic distance (km) among all populations are shown. To the left, COI results, and to the right, 18S results

The overall fixation index for *C. verrucosa* sp. A (COI: *F*_ST_ = 0.072; *p* < .05; 18S: *F*_ST_ = 0.154; *p* < .001) computed by AMOVA, pointed toward a high diversity and a strong structure among all WAP sites (results from pairwise *F*_ST_ genetic distance analysis for COI and 18S are shown in Table S2). Results from the Mantel test showed no correlation between geographic and genetic distance (*r* = .0165, *p* = .4510 for COI; and *r* = −0.0064, *p* = .4360 for 18S), see Figure 6.

On the other side, *C. verrucosa* sp. B showed no genetic structure among populations in COI sequences pairwise *F*_ST_ genetic distance analysis (Table S3), and sequences from 18S nuclear gene presented all the same haplotype. AMOVA overall fixation index for COI (COI: *F*_ST_ = 0.003; *p* > .05) also showed no genetic structure.

## 4 DISCUSSION

In the present study, we show that the conspicuous and widespread in the Antarctic ascidian *Cnemidocarpa verrucosa* comprises at least two genetically divergent species distributed in sympatry along the West Antarctic Peninsula. Moreover, results from Potter Cove population suggest that the basal disk could be a morphological character to differentiate the two species.

### 4.1 Two genetically divergent species

Both molecular markers studied in this work (the mitochondrial COI and nuclear 18S gene) distinguished two congruent groups; therefore, there was strong evidence for recognizing two genetically divergent species within *C. verrucosa* sensu lato from the WAP: *C. verrucosa* sp. A and *C. verrucosa* sp. B. Nuclear and mitochondrial genes evolve independently because they differ in the mode of inheritance, ploidy, amount of recombination, presence of introns, mutation rate, repair mechanisms, and effective population size (Hill, 2015). Thus, studying only one type of marker can lead to a systematic bias in the inference of evolutionary processes (Ballard & Whitlock, 2004; Seehausen et al., 2003). In this work, both markers showed the same speciation pattern, which implies that both molecules were impacted in the same way by the same evolutionary mechanism, thus making a strong case for the existence of mutually isolated gene pools.

Within the results obtained here, for some particular specimens no sequences were obtained of COI, while 18S nuclear gene was sequenced. In this case, the phylogeny based on nuclear 18S data resolved basal branches that comprised clades that would present deeper genetic divergence with the rest of the animals than the divergence among *C. verrucosa* sp. A and sp. B (see Figure 3). Nuclear genes usually evolve at a slower rate compared to mitochondrial ones (Allio, Donega, Galtier, & Nabholz, 2017; Havird & Sloan, 2016). Furthermore, fast substitution rate and gene rearrangements were described for ascidians mitochondrial genome and have been proposed to cause difficulties in standard polymerase chain reaction (PCR), because of mutations on the primer site (Delsuc et al., 2018; Denoeud et al., 2010; Gissi et al., 2010; Yokobori et al., 1999; Yokobori, Watanabe, & Oshima, 2003). Hereafter, the basal branches obtained in this study with 18S sequences and the groups C–F obtained in ABGD analysis for 18S were composed by individuals from MPAN‐BB, Scotia Sea, and Potter Cove (stations located in the tip of the WAP and South America; see Figure 1). These groups could represent even more cryptic species within *C. verrucosa* sensu lato and not accounted for in our sp. A and B, and be constituted by individuals in which the COI primer binding site has been mutated or rearranged.

Widely adopted molecular markers, such as COI and 18S, are helpful to characterize unstudied groups (Hebert, Cywinska, Ball, & Waard, 2003). Here, applying ABGD method in COI and 18S a barcode gap, with no intermediate values, was found in the frequency distribution of the genetic differences between individuals of the putative *C. verrucosa*. This gap is observed when the divergence between organisms that belong to the same species is smaller than the divergence among organisms that belong to different species (Puillandre et al., 2012). Moreover, a robust approach for species delimitation is to compare genetic distances with related undisputed species pairs, given that the nucleotide substitution rate is quite homogeneous at interspecific level (Griggio et al., 2014; Held, 2003). In this study, the genetic distance between *C. verrucosa* sp. A and sp. B was > 10.20% for COI, and > 0.11% for 18S. The COI nucleotide divergence among ascidian species from the same genera range from 10% to 24% (Nydam & Harrison, 2007; Pérez‐Portela & Turon, 2008), and between species within Styelidae family range from 10.8% to 16.5% (Lacoursière‐Roussel et al., 2012; Reem et al., 2017). Regarding the 18S gene, it has been found 0%–0.58% nucleotide divergence among samples from genera *Diplosoma*. (Yokobori, Kurabayashi, Neilan, Maruyama, & Hirose, 2006). Bock et al. (2012) found even larger divergences (2.3%–10.1%) in 18S gene among putative cryptic species of *Botryllus schlosseri*. The number and delimitation of cryptic species within *B. schlosseri* are still under discussion; therefore, *B. schlosseri* is currently being treated as a species complex (Lejeusne, Bock, Therriault, MacIsaac, & Cristescu, 2011; Nydam, Giesbrecht, et al., 2017; Reem et al., 2017; Yund et al., 2015). All this indicates that, within the samples studied here, the genetic differentiation was similar to those found in other species in the same family and other ascidians species pairs; therefore, we can define two genetic divergent species based on mitochondrial as well as nuclear evidence. Moreover, groups C, D, E, and F defined by ABGD analysis of 18S sequences showed divergences in the range shown by *B. schlosseri* species complex.

### 4.2 Two morphologically distinguishable species

The genera *Cnemidocarpa* belongs to the Styelidae (Ascidiacea) family (WoRMS, Shenkar et al., 2020) which is characterized by highly variable morphological characters (Monniot, Monniot, & Laboute, 1991). The genus *Cnemidocarpa* includes solitary ascidians with thin but leathery tunic; gonads that are elongated, tubular, and occasionally ramified, always united in a compact mass contained within a membrane and attached to the body wall (Kott, 1985; Rocha, Zanata, & Moreno, 2012). To distinguish the species within the genus, one of the most used characteristics is the number of gonads at each side of the body; however, *C. verrucosa* sensu lato presents a high variability from 1 to 4 gonads, and in this work, no significant variation was found in this character. A high variability of color and shape of warts was observed in the studied specimens and in the field (Figure 4), and no other new or already described characters were found to discriminate between genetically different species. However, we found that the presence/absence of basal disk could be a possible diagnostic character for identifying two genetic species in Potter Cove (where both species coexist): all *C. verrucosa* sp. A specimens lacked a basal disk, while all *C. verrucosa* sp. B had a well‐developed basal disk as described by Kott (1971). Tatian et al. (1998) already reported differences in stalk (basal disk in this study) development in *C. verrucosa* and *Molgula pedunculata* from Potter Cove. In their work, it was suggested that different substrate fixing requirements could determine the greater development of the stalk diameter in specimens of *C. verrucosa* from soft bottoms over those from hard bottoms. It was addressed in the literature that morphological differentiation of species depends on ecological/environmental factors and time since divergence (Fišer, Robinson, & Malard, 2018; Harmon et al., 2003, 2011; Losos, 2008; Schluter, 2000). Our results suggest that the well‐known morphological differentiation with regard to the presence or absence of the basal disk in *C. verrucosa* may not be a case of phenotypic plasticity in response to environmental conditions (e.g., bottom substrate) as previously assumed, but instead reflect a divergent genetical disposition of two reproductively isolated species. While it is still possible, even likely, that the presence of a basal disk may have an adaptive value (Givnish et al., 2014), our results strongly suggest that this is unlikely to lead to a flexible expression of phenotypic characters within the lifetime of an individual but instead exert its influence by determining the relative abundance of *C. verrucosa* sp. A or sp. B that either have or lack a basal disk in any given environment (see section 4.5). Nonetheless, to confirm this hypothesis it is necessary to address the morphological pattern of *C. verrucosa* sensu lato in a wider sampling range.

Morphological species delimitation is key, especially for recognition in the field when species are distributed in sympatry. *Cnemidocarpa verrucosa* was described initially by Lesson (1,830), and the type specimen was collected in Malvinas/Falklands Islands. In its original description and others works, the species was reported as possessing a high variability in shape, color, and size (Kott, 1971; Tatián, Antacli, & Sahade, 2005; Turon, Cañete, Sellanes, Rocha, & López‐legentil, 2016; Turon, Cañete, Sellanes, Rocha, & López‐Legentil, 2016); a pattern shared with other ascidians (Dias, Abreu, de Silva, & Solferini, 2008; Viard, Roby, Turon, Bouchemousse, & Bishop, 2019; Wiernes, Sahade, Tatián, & Chiappero, 2013). However, a character (morphological, molecular) may wrongly only appear to be polymorphic when two or more species are mistakenly treated as a single one. It is not uncommon that the apparent degree of polymorphism is strongly reduced once the cryptic or pseudocryptic species have been correctly identified (Dietz et al., 2015; Janosik & Halanych, 2010; Korshunova, Martynov, Bakken, & Picton, 2017; Montano, Maggioni, Galli, & Hoeksema, 2017).

### 4.3 Two species in sympatry

Species *C. verrucosa* sp. A and *C. verrucosa* sp. B are distributed in sympatry along the WAP. Both species are present in the Weddell Sea, Scotia Sea, Potter Cove, Shetland L45, Palmer Station, Paradise Bay, and Rothera Stations (Figure 1). The absence of *C. verrucosa* sp. B in some sampling stations may be explained by the low number of samples obtained on those sites. Even though depth differences that imply gradients in light, ice scouring, and other parameters could be a factor that determine species distribution, such as the case of an Antarctic polychaete (Schüller, 2011), a vertical zonation of sp. A and sp. B cannot be conclusively proven with our results since both species were found in deep and shallow sampling stations (see Table S1). Because both species coexist in sympatry but maintain genetic differences among them, a reproductive barrier must be playing an important role in keeping the species isolated. Broadcast spawners, like *C. verrucosa* sensu lato, release gametes into the water column where fertilization occurs; thus, the strength of the prezygotic reproductive barriers, like temporal isolation, can play a crucial role in reproductive isolation between species (Levitan et al., 2004). Another plausible hypothesis for reproductive isolation is gamete incompatibility, given that many studies on the evolution of gamete recognition proteins have shown that they tend to evolve more rapidly than other proteins, and frequently be under positive selection (Kosman & Levitan, 2014; Vacquier & Swanson, 2011). For example, in sea urchin species pairs, only 10 amino acid changes can lead to complete gamete incompatibility between species (Zigler, McCartney, Levitan, & Lessios, 2005). A clear example of closely related species living in sympatry which shows efficient reproductive barriers is *C. intestinalis* and *C. robusta*, and species in which natural hybridization has been shown to occur rarely (0.03%) and mitochondrial divergence among them (12%–14%) is comparable between *C. verrucosa* sp. A and B (10.20%). Furthermore, fertile hybrids in laboratory conditions were obtained among *C. intestinalis* and *C. robusta*, and in the field, sexually mature individuals producing gametes at the same time were observed, patterns that point toward the hypothesis that postzygotic reproductive barriers are playing an important role in this case (Bouchemousse, Bishop, & Viard, 2016; Bouchemousse, Liautard‐Haag, Bierne, & Viard, 2016; Nydam & Harrison, 2011; Sato, Shimeld, & Bishop, 2014).

The population structure of these species showed a striking and unexpected pattern, especially since they are largely sympatric and do not appear to have a strongly different dispersal potential. *C. verrucosa* sp. A showed genetically structured population, while no genetic structure was registered among the populations of *C. verrucosa* sp. B. Within *C. verrucosa* sp. A, IBD analysis showed no relation between genetic and geographic distance (Figure 6). *C. verrucosa* sensu lato presents a wide distribution range and with high registered abundances all around the Antarctic continent (Kott, 1969; Monniot et al., 2011; Tatián & Lagger, 2010; Tatian et al., 1998; Turon, Cañete, Sellanes, Rocha, & López‐legentil, 2016; Turon, Cañete, Sellanes, Rocha, & López‐Legentil, 2016); thus, the continuity and high abundance of populations could allow genetic connectivity, keeping an active gene flow over large distances. *Cnemidocarpa verrucosa* a priori disperse during the pelagic larval stage, which under laboratory conditions was described to last about 16 days, with 8 days as an unhatched embryo and up to 8 or more days as a tadpole (Strathmann et al., 2006). Thus, transport of larvae can be helped by set of inshore currents described along the west Antarctic coast of the peninsula that moves the water along a large cyclonic gyre with some cyclonic subgyres (Moffat & Meredith, 2018; Smith, Hofmann, Klinck, & Lascara, 1999). In the absence of more specific knowledge, it may be hypothesized that *C. verrucosa* sp. B has a more continuous distribution pattern along its distribution range allowing a higher gene flow, while *C. verrucosa* sp. A presents discrete populations with more restricted gene flow. Indeed, it has been suggested for ascidians that high mutation rates in both the nuclear and the mitochondrial genomes enable the accumulation of genetic diversity in relatively isolated populations (Delsuc, Brinkmann, Chourrout, & Philippe, 2006; Reem, Douek, Katzir, & Rinkevich, 2013), process that can explain IBD pattern for *C. verrcuosa* sp. A. On top of this, sp. B may have dispersed in the area more recently, having no time to accumulate genetic diversity, and/or may have spread from a more homogeneous source than sp. A. Finally, the observed patter could be related to different capabilities for colonizing different substrate types and/or to differential dispersal potential among the two species. Thus, the reported capability of inhabiting all substrates of *C. verrucosa* sensu lato (Ramos‐Esplá et al., 2005; Tatian et al., 1998) would be mainly a *C. verrucosa* sp. B trait, and this species could present a longer larval stage and dispersal potential than its counterpart *C. verrucosa* sp. A. Despite being speculative hypothesis, they drive the attention to the fact that the reported variability on many biological and ecological traits for *C. verrucosa* sensu lato can be due to added characteristics of two species rather than an actual variability of the species.

### 4.4 Incongruent mitochondrial/nuclear pattern

An interesting pattern was observed when species delimitation was performed with both markers: two individuals were not assigned to the same group for both genes (we resequenced both genes and obtained unchanged results for both individuals). On the one hand, individual 291 was assigned to *C. verrucosa* sp. A in 18S species delimitation but to *C. verrucosa* sp. B for COI; on the other hand, individual 116 was assigned to *C. verrucosa* sp. B in 18S species delimitation but to *C. verrucosa* sp. A for COI. In both these cases, the incongruent assignment to different taxonomic groups depending on the data source (mitochondrial or nuclear gene) was not due to a lack of resolution in either one of them, but instead by possessing the character state in the only informative 18S position (see section 3.1) that would be expected for the species that the mitochondrial COI gene suggested the individual concerned did not belong to. Apparent incongruence between phylogenies from 18S and COI sequences was addressed already in ascidians by other authors (Pérez‐Portela, Bishop, Davis, & Turon, 2009; Stach & Turbeville, 2002). In this study, while *C. verrucosa* sp. A was characterized by a thymine in the unique variable site of 18S sequences, *C. verrucosa* sp. B was characterized by a cytosine. Specimen 291 had two overlapping peaks (thymine and cytosine) at this site. Both *C. verrucosa* sp. A and sp. B may therefore retain rare alleles of the character state typical for their sister species as a consequence of an ancestral polymorphism at this site (ancestral polymorphism with incomplete linages sorting, ILS), and individual 291 would be a heterozygote according to this interpretation (Hoy & Rodriguez, 2013; Rooney, 2004; Shapoval & Lukhtanov, 2015). Alternatively, the pattern could also be explained by low occurrence of hybridization between *C. verrucosa* sp. A and sp. B.

Hybridization and introgression were already proposed in ascidians (Bouchemousse, Liautard‐Haag, et al., 2016; Nydam, Yanckello, et al., 2017). Broadcast spawners present a reproductive system that has a higher probability of introgression/hybridization than others. While artificial cross‐fertilization among *Ciona intestinalis* type A and B was demonstrated, hybrids remained infertile (Caputi et al., 2007; Sato et al., 2014). Under those circumstances, it would be of interest to experimentally test hybridization among *C. verrucosa* sp. A and sp. B. Furthermore, it is difficult to distinguish ILS from introgression since both produce similar patterns of discrepancies between trees inferred with mitochondrial and nuclear genes (Zhou et al., 2017). There is circumstantial evidence that ascidians may be characterized by an elevated rate of molecular evolution (Delsuc et al., 2006). Therefore, a convergent mutation can also explain that individual 116 presents allele B in 18S, but being assigned to *C. verrucosa* sp. A for COI, given that the thymine mutates to a cytosine in the unique variable site.

### 4.5 Timing and mode of speciation

In order to estimate time since speciation, data from crabs, shrimps, and urchins were used as proxy (mutation rate range of 0.016–0.026 substitutions per site, per million years) (Nydam & Harrison, 2011). Using these rate estimates, we obtained the speciation of *C. verrucosa* sp. A and sp. B at 3.58–2.20 million years ago (MYA). Several estimations of whole‐genome mutation rates have been calculated in ascidians pointing out the rapid evolution of this group (Berna & Alvarez‐Valin, 2014; Denoeud et al., 2010), but not specifically for mitochondrial genomes, which typically evolve faster than the nuclear genome (Havird & Sloan, 2016). On average, the substitution rate in ascidians is 6.25 times faster than in vertebrates and 2.08 faster than in cephalochordates (Delsuc et al., 2018). This indicates that, even though we can estimate the speciation time based on other marine invertebrate taxa data, we should bear in mind that we may be overestimating the time since divergence and thus the actual speciation time likely being more recent. We can hypothesize that speciation took place after the Miocene, when Antarctica already experienced the cooling process (Zachos, Pagani, Sloan, Thomas, & Billups, 2001). Many other studies reported radiation and speciation processes around 8–5 MYA; and cycles of population concentration, isolation in refugia and expansion, speciation, and transoceanic dispersal by 1 MYA (Rogers, 2007). Some examples of these processes in Antarctic taxa are arthropods, annelids, echinoderms, and molluscs (Baird, Miller, & Stark, 2011; Hemery et al., 2012; Linse, Cope, Lörz, & Sands, 2007; Raupach et al., 2010; Riesgo et al., 2015; Wilson et al., 2007). Then, a similar pattern of allopatric speciation followed by secondary contact (Mayr, 1963) can be attributed to *C. verrucosa*. On the other hand, speciation in response to ecological opportunity (Simpson, 1953) can also be hypothesized with our results. Under this type of speciation, a new trait evolves and affects the ecological versatility of the specimens (Givnish et al., 2014; Liem, 1973). In this case, the development of a basal disk could represent an adaptive character for colonizing different substrates.

## 5 CONCLUSIONS

This work provides new insights to understand the evolution of molecules and morphology at the same time. The evidence presented here allowed to recognize two species within *C. verrucosa*: two monophyletic groups were obtained; congruent mitochondrial, nuclear species delimitation was demonstrated. The magnitude of difference between the two distinguished species was similar to those among undisputed species pairs, and both species were distributed in sympatry. Morphological analysis suggests that the presence of a basal disk could be a morphological feature distinguishing the species. Nominal *C. verrucosa* sensu lato are distributed all around Antarctica and in the south of South America. However, the true extent of the species distribution area is obscured by the existence of two, possibly more species that have up until now been mistaken as a single species. Incongruent mitochondrial–nuclear phylogenies might be explained by different hypotheses as introgression/hybridization or ILS. Boundaries between sympatric species are maintained by barriers to gene flow, and these restrictions may not be uniform in space, time, or across the genome. In fact, it has been proposed that these barriers are semipermeable and speciation under gene flow is possible (Nosil, 2008). Further analysis employing several nuclear loci and an extended geographic sampling would help to elucidate the evolutionary story of this broadly distributed Antarctic ascidian species. Patterns regarding genetic and morphological differentiation that are being underestimated or not registered systematically can lead to important misunderstanding of species distribution patterns related to adaptation, habitat preference, competition, and response to climate change. Our results once again emphasize that species identities, even for highly abundant and well‐studied species on small local scales, must be assessed rather than assumed.

## CONFLICT OF INTEREST

The authors declare that they have no conflicts of interest.

## AUTHOR CONTRIBUTION

**Micaela Belen Ruiz:** Conceptualization (lead); Formal analysis (lead); Investigation (equal); Methodology (lead); Writing‐original draft (lead); Writing‐review & editing (lead). **Anabela Taverna:** Methodology (equal); Writing‐review & editing (supporting). **Natalia Servetto:** Investigation (equal); Methodology (equal); Writing‐review & editing (equal). **Ricardo Sahade:** Conceptualization (lead); Funding acquisition (lead); Investigation (equal); Project administration (lead); Supervision (equal); Writing‐review & editing (equal). **Christoph Held:** Conceptualization (lead); Formal analysis (supporting); Funding acquisition (equal); Investigation (equal); Methodology (equal); Project administration (equal); Resources (lead); Supervision (lead); Writing‐review & editing (equal).

## Supporting information

TableS1

TableS2

TableS3

## Data Availability

Sequence data can be found in NCBI GenBank (https://www.ncbi.nlm.nih.gov/genbank/), 18S entire sequences with accession numbers MN700311‐MN700622, COI entire sequences and protein traduction with accession numbers MN714370‐MN714622. Morphological and genetic data can be found in PANGEA (https://www.pangaea.de/), https://doi.org/10.1594/PANGAEA.909707.
