# Supplementary material for: Hidden diversity in Antarctica: Molecular and morphological evidence of two different species within one of the most conspicuous ascidian species
Source: Ecol Evol. 2020 Jul 15;10(15):8127–43. doi: 10.1002/ece3.6504 (PMC7417227; doi:10.1002/ece3.6504)
Supplement: Supplementary file 1 — TableS1 [file ECE3-10-8127-s001.docx]

| **Sampling site** | **Depth (m)** | **COI** | | | | **18S** | | | |
| --- | --- | --- | --- | --- | --- | --- | --- | --- | --- |
|  |  | **N** | **h** | **Hd** | **π** | **N** | **h** | **Hd** | **π** |
| Burdwood Bank/MPA Namuncurá | 91-460 | 1 | 1 | 0.000±0.000 | 0.00000±0.00000 | 7 | 3 | 0.659±0.072 | 0.01174±0.00573 |
| Potter Cove | 20-30 | 102 | 18 | 0.783±0.026 | 0.05027± 0.00156 | 115 | 5 | 0.539±0.015 | 0.00200±0.00065 |
| Livingston Island | 20-30 | 8 | 2 | 0.429±0.169 | 0.00256±0.00101 | 16 | 1 | 0.000±0.000 | 0.00000±0.00000 |
| Scotia Sea | 171-428 | 7 | 2 | 0.286±0.196 | 0.00170±0.00117 | 25 | 4 | 0.566±0.065 | 0.00916±0.00182 |
| Shetland L45 | 170-179 | 3 | 2 | 0.667±0.314 | 0.00530±0.00250 | 8 | 3 | 0.633±0.074 | 0.00084±0.00015 |
| Shetland L46 | 120-129 | 4 | 2 | 0.500±0.265 | 0.00497±0.00264 | 6 | 2 | 0.545±0.062 | 0.00062±0.00007 |
| Weddell Sea | 217-375 | 16 | 4 | 0.517±0.132 | 0.03986±0.01124 | 37 | 3 | 0.394±0.060 | 0.00054±0.00009 |
| Mikkelsen Island | 20-30 | 7 | 3 | 0.667±0.160 | 0.00341±0.00102 | 5 | 1 | 0.000±0.000 | 0.00000±0.00000 |
| Charlotte Bay | 20-30 | 2 | 1 | 0.000±0.000 | 0.00000±0.00000 | 2 | 1 | 0.000±0.000 | 0.00000±0.00000 |
| Melchior Island | 20-30 | 6 | 3 | 0.733±0.155 | 0.00225±0.00049 | 6 | 1 | 0.000±0.000 | 0.00000±0.00000 |
| Palmer Station | 20-30 | 18 | 7 | 0.739±0.099 | 0.03812±0.01106 | 18 | 2 | 0.286±0.084 | 0.00033±0.00010 |
| Paradise Bay | 20-30 | 11 | 4 | 0.600±0.154 | 0.02057±0.01376 | 11 | 2 | 0.173±0.101 | 0.00020±0.00012 |
| Fish Island | 20-30 | 15 | 5 | 0.705±0.088 | 0.00386±0.00053 | 15 | 1 | 0.000±0.000 | 0.00000±0.00000 |
| Renaud Island | 20-30 | 11 | 3 | 0.691± 0.086 | 0.00390±0.00050 | 11 | 1 | 0.000±0.000 | 0.00000±0.00000 |
| Rothera Station | 20-30 | 42 | 6 | 0.602±0.043 | 0.01209±0.00568 | 37 | 3 | 0.177±0.058 | 0.00021±0.00007 |

Table S1. Sampling stations and depth of sampling. Genetic polymorphism analysis was run for each population calculating the number of haplotypes (Nh), haplotype diversity (h) and nucleotide diversity (π) using DnaSP 5.10 (Librado & Rozas, 2009).
