# Supplementary material for: Hidden diversity in Antarctica: Molecular and morphological evidence of two different species within one of the most conspicuous ascidian species
Source: Ecol Evol. 2020 Jul 15;10(15):8127–43. doi: 10.1002/ece3.6504 (PMC7417227; doi:10.1002/ece3.6504)
Supplement: Supplementary file 2 — TableS2 [file ECE3-10-8127-s002.docx]

|  | Potter Cove | Palmer Station | Rothera Station | Paradise Bay | Shetland L45 | Scotia Sea | Weddell Sea | Melchior Island | Charlotte Bay | Mikkelsen Island | Fish Island | Renaud Island | Livingston Island | Shetland L46 |
| --- | --- | --- | --- | --- | --- | --- | --- | --- | --- | --- | --- | --- | --- | --- |
| Potter Cove | 0 | -0.066 | -0.008 | -0.001 | **0.406** | **0.064** | **0.108** | -0.019 | -0.116 | -0.019 | 0.010 | 0.002 | -0.007 | **0.501** |
| Palmer Station | -0.103 | 0 | -0.055 | 0 | 0.266 | 0.027 | 0.060 | 0 | 0 | 0 | 0 | 0 | 0 | 0.351 |
| Rothera Station | -0.017 | -0.067 | 0 | 0.014 | **0.386** | 0.046 | 0.089 | -0.006 | -0.103 | -0.006 | 0.027 | 0.018 | 0.006 | **0.486** |
| Paradise Bay | -0.036 | -0.196 | -0.014 | 0 | **0.443** | 0.114 | **0.189** | 0 | 0 | 0 | 0 | 0 | 0 | **0.542** |
| Shetland L45 | -0.116 | -0.290 | -0.096 | -0.226 | 0 | 0.103 | 0.033 | **0.361** | 0.211 | **0.361** | **0.517** | **0.460** | **0.406** | -0.073 |
| Scotia Sea | **0.198** | 0.019 | **0.259** | 0.114 | 0.097 | 0 | -0.038 | 0.081 | -0.020 | 0.081 | **0.142** | **0.120** | 0.099 | 0.180 |
| Weddell Sea | 0.065 | -0.143 | 0.132 | -0.031 | -0.044 | -0.030 | 0 | 0.132 | 0.006 | 0.132 | **0.242** | **0.201** | 0.163 | 0.097 |
| Melchior Island | 0.130 | 0.026 | 0.191 | 0.095 | 0.048 | 0.2 | -0.015 | 0 | 0 | 0 | 0 | 0 | 0 | **0.454** |
| Charlotte Bay | 0.031 | -0.263 | 0.109 | -0.118 | -0.2 | -0.304 | -0.263 | 0.047 | 0 | 0 | 0 | 0 | 0 | 0.294 |
| Mikkelsen Island | 0.094 | 0.173 | 0.054 | 0.161 | 0.003 | **0.505** | 0.433 | **0.402** | 0.423 | 0 | 0 | 0 | 0 | **0.454** |
| Fish Island | 0.030 | 0.044 | 0.002 | 0.073 | -0.035 | **0.361** | 0.258 | **0.274** | 0.242 | -0.070 | 0 | 0 | 0 | **0.617** |
| Renaud Island | -0.032 | -0.093 | -0.039 | -0.054 | -0.219 | 0.242 | 0.115 | 0.169 | 0.066 | -0.008 | -0.023 | 0 | 0 | **0.559** |
| Livingston Island | 0.131 | 0.269 | 0.089 | 0.236 | 0.143 | **0.578** | 0.529 | **0.478** | 0.543 | -0.133 | -0.051 | 0.063 | 0 | **0.502** |
| Shetland L46 | **0.243** | 0 | **0.290** | 0.130 | 0.003 | 0.043 | 0 | 0.167 | -0.263 | **0.427** | **0.346** | 0.226 | **0.506** | 0 |

Table S2. Pairwise *F_ST_* among all sampling stations of *Cnemidocarpa verrucosa* sp. A. In the upper side of the diagonal values correspond to 18S sequences, the lower side of the diagonal values correspond COI sequences. Numbers in bold indicate significant results (p<0.05).
