# Supplementary material for: Hidden diversity in Antarctica: Molecular and morphological evidence of two different species within one of the most conspicuous ascidian species
Source: Ecol Evol. 2020 Jul 15;10(15):8127–43. doi: 10.1002/ece3.6504 (PMC7417227; doi:10.1002/ece3.6504)
Supplement: Supplementary file 3 — TableS3 [file ECE3-10-8127-s003.docx]

|  | Potter Cove | Palmer Station | Rothera Station | Weddell Sea |
| --- | --- | --- | --- | --- |
| Potter Cove | 0.000 |  |  |  |
| Palmer Station | -0.003 | 0.000 |  |  |
| Rothera Station | 0.129 | -0.008 | 0.000 |  |
| Weddell Sea | 0.016 | 0.033 | 0.508 | 0.000 |

Table S3. Pairwise COI-*F_ST_* among sampling stations of *Cnemidocarpa verrucosa* sp. B. No significant (p<0.05) values were found.
